# The genome sequence of the smooth giant clam, *Tridacna derasa *Röding, 1798

**DOI:** 10.12688/wellcomeopenres.22618.1

**Published:** 2024-07-16

**Authors:** Ruiqi Li, Jingchun Li, Jose Victor Lopez, Graeme Oatley, Isabelle Ailish Clayton-Lucey, Elizabeth Sinclair, Eerik Aunin, Noah Gettle, Camilla Santos, Michael Paulini, Haoyu Niu, Victoria McKenna, Rebecca O’Brien, Wellcome Sanger Institute Tree of Life Management, Samples and Laboratory Team, Wellcome Sanger Institute Scientific Operations: Sequencing Operations, Wellcome Sanger Institute Tree of Life Core Informatics Team, EBI Aquatic Symbiosis Genomics Data Portal Team, Aquatic Symbiosis Genomics Project Leadership

**Affiliations:** 1 Ecology & Evolutionary Biology, University of Colorado Boulder, Boulder, Colorado, USA; 2 University of Colorado Boulder Museum of Natural History, Boulder, Colorado, USA; 3 Nova Southeastern University, Dania Beach, Florida, USA; 4 Wellcome Sanger Institute, Hinxton, England, UK

**Keywords:** Tridacna derasa, smooth giant clam, genome sequence, chromosomal, Cardiida; Tridacnidae

## Abstract

We present a genome assembly from an individual *Tridacna derasa* (the smooth giant clam; Mollusca; Bivalvia;Cardiida; Cardiidae). The genome sequence is 1,060.2 megabases in span. Most of the assembly is scaffolded into 18 chromosomal pseudomolecules. The mitochondrial genome has also been assembled and is 24.95 kilobases in length. Gene annotation of this assembly on Ensembl identified 19,638 protein coding genes.

## Species taxonomy

Eukaryota; Opisthokonta; Metazoa; Eumetazoa; Bilateria; Protostomia; Spiralia; Lophotrochozoa; Mollusca; Bivalvia; Autobranchia; Heteroconchia; Euheterodonta; Imparidentia; Neoheterodontei; Cardiida; Cardioidea; Cardiidae; Tridacninae; *Tridacna*; *Tridacna derasa* Röding, 1798 (NCBI:txid80831).

## Background

The smooth giant clam, *Tridacna derasa* (subfamily: Tridacninae), is the second largest giant clam species. Like other species in the subfamily, *T. derasa* engages in an obligate symbiotic relationship with Symbiodiniaceae dinoflagellates ( Ip & Chew, 2021). Spanning a vast range from the Cocos Islands to Tonga and from China to Queensland, Australia, *T. derasa* dwells in varied tropical environments, including reef flats, lagoons, at depths of up to 20 metres ( Neo, 2023). Phylogenetically, *T. derasa* occupies a distinct position within the Tridacninae ( Tan *et al.*, 2022). Compared to the early-diverging species, such as *T. gigas*, it has unique morphological features, including the presence of guard tentacles on its incurrent siphon ( Tan *et al.*, 2022). Investigating *T. derasa’*s genomic foundations can shed light on the determinants of its large size, symbiotic efficiency, and adaptability to a wide array of marine environments, offering vital information for the conservation of this species and the coral reef ecosystems they inhabit. Moreover, genetic analyses have identified significant differentiation among *T. derasa* populations across regions like the Great Barrier Reef, Fiji, and the Philippines, unlike *T. maxima* ( Macaranas *et al.*, 1992) *.* Comprehensive genome analysis will enhance our understanding of the species’ complex population dynamics and genetic diversity, which will aid for conservation of unique populations under the changing marine environments. Together with genomes from other genera in the subfamily ( Li *et al*,. 2023; Li *et al*., 2024a; Li *et al*., 2024b), it provides a comprehensive framework to study genome evolution in the giant clams, which can uncover patterns of genetic divergence and convergence in the giant clam genome evolution. 

## Genome sequence report

**Figure 1.**
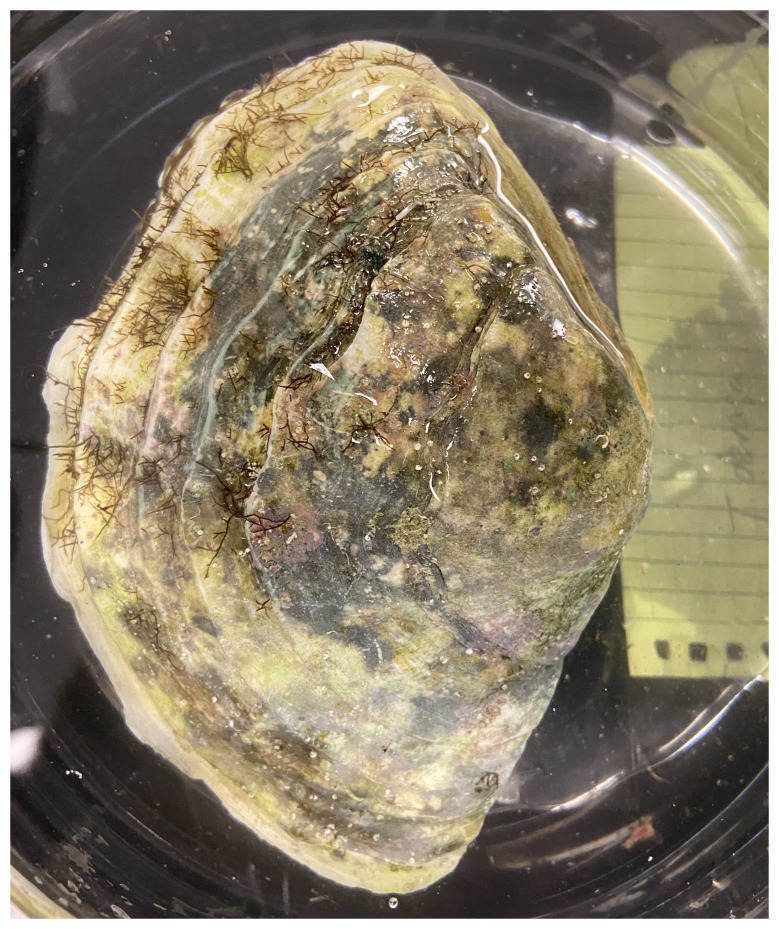
Photograph of the *Tridacna derasa* (xbTriDera4) specimen used for genome sequencing.

The genome was sequenced from *Tridacna derasa* ( Figure 1) collected from Marshall Islands Mariculture Farm, Majuro, Marshall Islands. A total of 49-fold coverage in Pacific Biosciences single-molecule HiFi long reads was generated. Primary assembly contigs were scaffolded with chromosome conformation Hi-C data. Manual assembly curation corrected 32 missing joins or mis-joins and removed 6 haplotypic duplications, reducing the assembly length by 0.71% and increasing the scaffold number by 4.76%.

**Figure 2.**
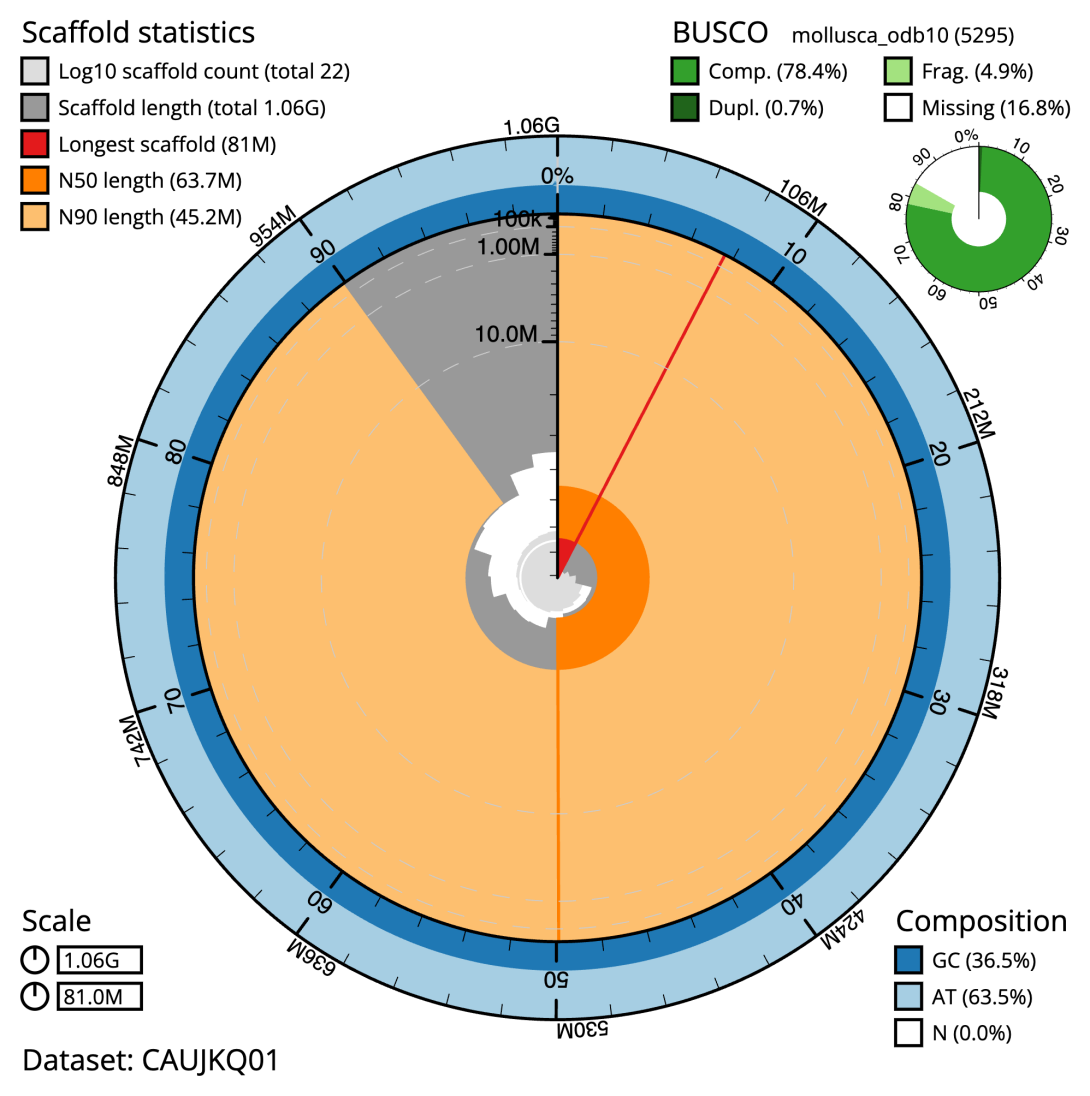
Genome assembly of *Tridacna derasa*, xbTriDera4.1: metrics. The BlobToolKit snail plot shows N50 metrics and BUSCO gene completeness. The main plot is divided into 1,000 size-ordered bins around the circumference with each bin representing 0.1% of the 1,060,251,317 bp assembly. The distribution of sequence lengths is shown in dark grey with the plot radius scaled to the longest sequence present in the assembly (81,017,200 bp, shown in red). Orange and pale-orange arcs show the N50 and N90 sequence lengths (63,680,613 and 45,204,528 bp), respectively. The pale grey spiral shows the cumulative sequence count on a log scale with white scale lines showing successive orders of magnitude. The blue and pale-blue area around the outside of the plot shows the distribution of GC, AT and N percentages in the same bins as the inner plot. A summary of complete, fragmented, duplicated and missing BUSCO genes in the mollusca_odb10 set is shown in the top right. An interactive version of this figure is available at https://blobtoolkit.genomehubs.org/view/CAUJKQ01/dataset/CAUJKQ01/snail.

**Figure 3.**
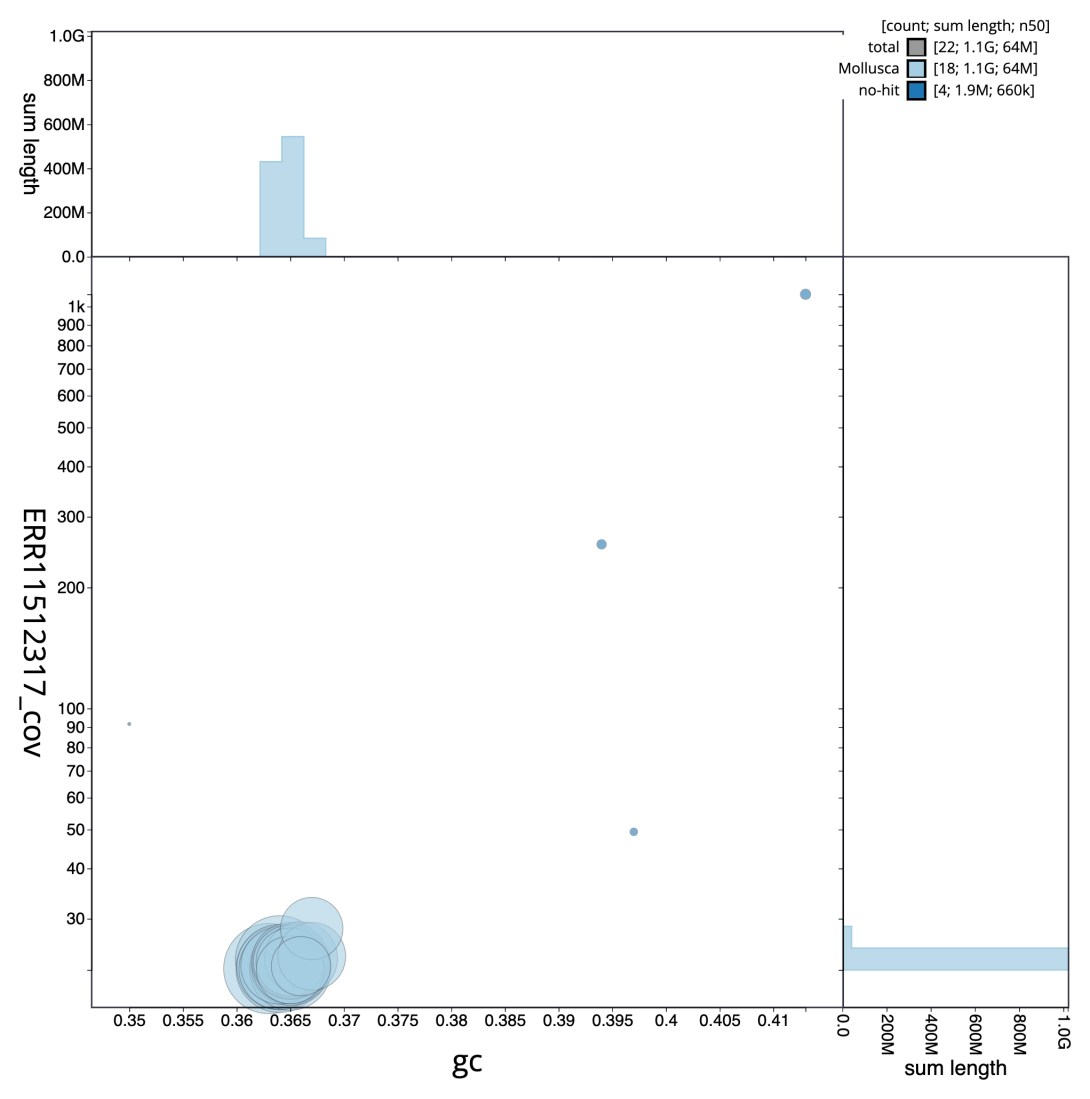
Genome assembly of *Tridacna derasa*, xbTriDera4.1: BlobToolKit GC-coverage plot. Scaffolds are coloured by phylum. Circles are sized in proportion to scaffold length. Histograms show the distribution of scaffold length sum along each axis. An interactive version of this figure is available at https://blobtoolkit.genomehubs.org/view/CAUJKQ01/dataset/CAUJKQ01/blob.

**Figure 4.**
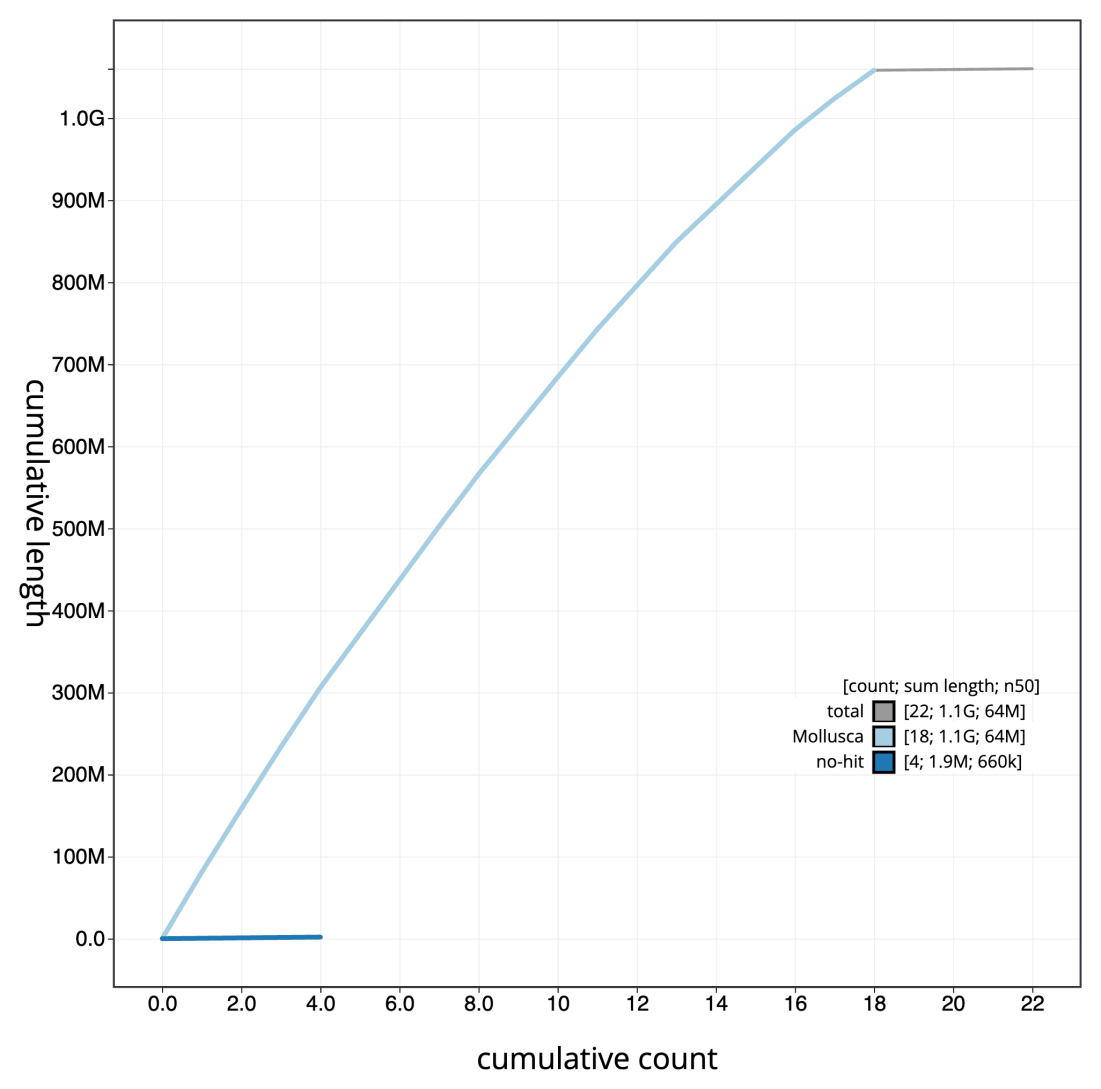
Genome assembly of *Tridacna derasa*, xbTriDera4.1: BlobToolKit cumulative sequence plot. The grey line shows cumulative length for all scaffolds. Coloured lines show cumulative lengths of scaffolds assigned to each phylum using the buscogenes taxrule. An interactive version of this figure is available at https://blobtoolkit.genomehubs.org/view/CAUJKQ01/dataset/CAUJKQ01/cumulative.

**Figure 5.**
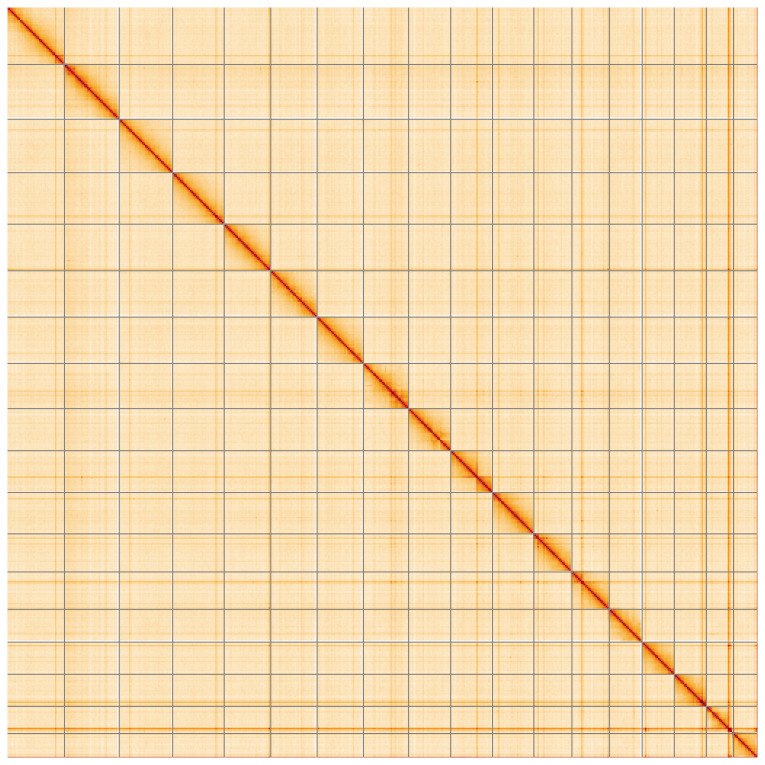
Genome assembly of *Tridacna derasa*, xbTriDera4.1: Hi-C contact map of the xbTriDera4.1 assembly, visualised using HiGlass. Chromosomes are shown in order of size from left to right and top to bottom. An interactive version of this figure may be viewed at https://genome-note-higlass.tol.sanger.ac.uk/l/?d=MGzvx7ywRIiSlJLvKCRZkg.

The final assembly has a total length of 1060.2 Mb in 21 sequence scaffolds with a scaffold N50 of 63.7 Mb ( Table 1). The snail plot in Figure 2 provides a summary of the assembly statistics, while the distribution of assembly scaffolds on GC proportion and coverage is shown in Figure 3. The cumulative assembly plot in Figure 4 shows curves for subsets of scaffolds assigned to different phyla. Most (99.82%) of the assembly sequence was assigned to 18 chromosomal-level scaffolds. Chromosome-scale scaffolds confirmed by the Hi-C data are named in order of size ( Figure 5; Table 2). While not fully phased, the assembly deposited is of one haplotype. Contigs corresponding to the second haplotype have also been deposited. The mitochondrial genome was also assembled and can be found as a contig within the multifasta file of the genome submission.

**Table 1.** Genome data for *Tridacna derasa*, xbTriDera4.1.

Project accession data		
Assembly identifier	xbTriDera4.1	
Species	*Tridacna derasa*	
Specimen	xbTriDera4	
NCBI taxonomy ID	80831	
BioProject	PRJEB62732	
BioSample ID	SAMEA8576963	
Isolate information	xbTriDera4 (DNA, Hi-C and RNA sequencing)	
Assembly metrics *		*Benchmark*
Consensus quality (QV)	68.3	*≥ 50*
*k*-mer completeness	100.0%	*≥ 95%*
BUSCO **	C:78.4%[S:77.6%,D:0.7%], F:4.9%,M:16.8%,n:5,295	*C ≥ 95%*
Percentage of assembly mapped to chromosomes	99.82%	*≥ 95%*
Sex chromosomes	None	*localised * *homologous pairs*
Organelles	Mitochondrial genome: 24.95 kb	*complete single * *alleles*
Raw data accessions		
PacificBiosciences SEQUEL II	ERR11512317, ERR11512318, ERR11512319	
Hi-C Illumina	ERR11526207, ERR11526206	
PolyA RNA-Seq Illumina	ERR11526208	
Genome assembly		
Assembly accession	GCA_963210305.1	
*Accession of alternate * *haplotype*	GCA_963210365.1	
Span (Mb)	1,060.2	
Number of contigs	394	
Contig N50 length (Mb)	4.7	
Number of scaffolds	21	
Scaffold N50 length (Mb)	63.7	
Longest scaffold (Mb)	81.02	

* Assembly metric benchmarks are adapted from column VGP-2020 of “Table 1: Proposed standards and metrics for defining genome assembly quality” from ( Rhie *et al.*, 2021). ** BUSCO scores based on the mollusca_odb10 BUSCO set using version 5.3.2. C = complete [S = single copy, D = duplicated], F = fragmented, M = missing, n = number of orthologues in comparison. A full set of BUSCO scores is available at https://blobtoolkit.genomehubs.org/view/CAUJKQ01/dataset/CAUJKQ01/busco.

The estimated Quality Value (QV) of the final assembly is 68.3 with *k*-mer completeness of 100.0%, and the assembly has a BUSCO v5.3.2 completeness of 78.4% (single = 77.6%, duplicated = 0.7%), using the mollusca_odb10 reference set ( *n* = 5,295).

Metadata for specimens, barcode results, spectra estimates, sequencing runs, contaminants and pre-curation assembly statistics are given at https://links.tol.sanger.ac.uk/species/80831.

**Table 2.** Chromosomal pseudomolecules in the genome assembly of *Tridacna derasa*, xbTriDera4.

INSDC accession	Chromosome	Length (Mb)	GC%
OY723414.1	1	81.02	36.5
OY723415.1	2	77.16	36.5
OY723416.1	3	75.31	36.5
OY723417.1	4	72.64	36.5
OY723418.1	5	65.62	36.5
OY723419.1	6	65.32	36.5
OY723420.1	7	65.29	36.5
OY723421.1	8	63.68	36.5
OY723422.1	9	59.29	36.5
OY723423.1	10	58.89	36.5
OY723424.1	11	58.37	36.5
OY723425.1	12	53.67	36.5
OY723426.1	13	52.69	36.5
OY723427.1	14	46.22	36.5
OY723428.1	15	45.37	36.5
OY723429.1	16	45.2	36.5
OY723430.1	17	38.19	36.5
OY723431.1	18	34.4	36.5
OY723432.1	MT	0.02	35.0

## Genome annotation report

The *Tridacna derasa* genome assembly (GCA_963210305.1) was annotated at the European Bioinformatics Institute (EBI) on Ensembl Rapid Release. The resulting annotation includes 37,937 transcribed mRNAs from 19,638 protein-coding and 7,310 non-coding genes ( Table 2; https://rapid.ensembl.org/Tridacna_derasa_GCA_963210305.1/Info/Index).

## Methods

### Sample acquisition and nucleic acid extraction

A *Tridacna derasa* (specimen ID NSU0010201, ToLID xbTriDera4) was purchased from Oceans, Reefs & Aquariums (ORA) in Marshall Islands Mariculture Farm, Majuro, Marshall Islands. The specimen was collected and identified by Jingchun Li and Ruiqi Li (University of Colorado Boulder), and then preserved by snap-freezing.

The workflow for high molecular weight (HMW) DNA extraction at the Wellcome Sanger Institute (WSI) includes a sequence of core procedures: sample preparation; sample homogenisation, DNA extraction, fragmentation, and clean-up. In sample preparation, the xbTriDera4 sample was weighed and dissected on dry ice ( Jay *et al.*, 2023). For sample homogenisation, tissue was cryogenically disrupted using the Covaris cryoPREP ^®^ Automated Dry Pulverizer ( Narváez-Gómez *et al.*, 2023).

HMW DNA was extracted using the Manual MagAttract v1 protocol ( Strickland *et al.*, 2023b). DNA was sheared into an average fragment size of 12–20 kb in a Megaruptor 3 system with speed setting 30 ( Todorovic *et al.*, 2023). Sheared DNA was purified by solid-phase reversible immobilisation ( Strickland *et al.*, 2023a): in brief, the method employs a 1.8X ratio of AMPure PB beads to sample to eliminate shorter fragments and concentrate the DNA. The concentration of the sheared and purified DNA was assessed using a Nanodrop spectrophotometer and Qubit Fluorometer and Qubit dsDNA High Sensitivity Assay kit. Fragment size distribution was evaluated by running the sample on the FemtoPulse system.

RNA was extracted from xbTriDera4 in the Tree of Life Laboratory at the WSI using the RNA Extraction: Automated MagMax™ *mir*Vana protocol ( do Amaral *et al.*, 2023). The RNA concentration was assessed using a Nanodrop spectrophotometer and a Qubit Fluorometer using the Qubit RNA Broad-Range Assay kit. Analysis of the integrity of the RNA was done using the Agilent RNA 6000 Pico Kit and Eukaryotic Total RNA assay.

Protocols developed by the WSI Tree of Life laboratory are publicly available on protocols.io ( Denton *et al.*, 2023).

### Sequencing

Pacific Biosciences HiFi circular consensus DNA sequencing libraries were constructed according to the manufacturers’ instructions. Poly(A) RNA-Seq libraries were constructed using the NEB Ultra II RNA Library Prep kit. DNA and RNA sequencing was performed by the Scientific Operations core at the WSI on Pacific Biosciences SEQUEL II (HiFi) and Illumina NovaSeq 6000 (RNA-Seq) instruments. Hi-C data were also generated from tissue of xbTriDera4 using the Arima2 kit and sequenced on the Illumina NovaSeq 6000, Illumina NovaSeq 6000 instrument.

### Genome assembly, curation and evaluation

Assembly was carried out with Hifiasm ( Cheng *et al.*, 2021) and haplotypic duplication was identified and removed with purge_dups ( Guan *et al.*, 2020). The assembly was then scaffolded with Hi-C data ( Rao *et al.*, 2014) using YaHS ( Zhou *et al.*, 2023). The assembly was checked for contamination and corrected using the gEVAL system ( Chow *et al.*, 2016) as described previously ( Howe *et al.*, 2021). Manual curation was performed using gEVAL, HiGlass ( Kerpedjiev *et al.*, 2018) and Pretext ( Harry, 2022). The mitochondrial genome was assembled using MitoHiFi ( Uliano-Silva *et al.*, 2023), which runs MitoFinder ( Allio *et al.*, 2020) or MITOS ( Bernt *et al.*, 2013) and uses these annotations to select the final mitochondrial contig and to ensure the general quality of the sequence.

A Hi-C map for the final assembly was produced using bwa-mem2 ( Vasimuddin *et al.*, 2019) in the Cooler file format ( Abdennur & Mirny, 2020). To assess the assembly metrics, the *k*-mer completeness and QV consensus quality values were calculated in Merqury ( Rhie *et al.*, 2020). This work was done using Nextflow ( Di Tommaso *et al.*, 2017) DSL2 pipelines “sanger-tol/readmapping” ( Surana *et al.*, 2023a) and “sanger-tol/genomenote” ( Surana *et al.*, 2023b). The genome was analysed within the BlobToolKit environment ( Challis *et al.*, 2020) and BUSCO scores ( Manni *et al.*, 2021; Simão *et al.*, 2015) were calculated.

Table 3 contains a list of relevant software tool versions and sources.

**Table 3.** Software tools: versions and sources.

Software tool	Version	Source
BlobToolKit	4.2.1	https://github.com/blobtoolkit/blobtoolkit
BUSCO	5.3.2	https://gitlab.com/ezlab/busco
Hifiasm	0.16.1	https://github.com/chhylp123/hifiasm
HiGlass	1.11.6	https://github.com/higlass/higlass
Merqury	MerquryFK	https://github.com/thegenemyers/MERQURY.FK
MitoHiFi	2	https://github.com/marcelauliano/MitoHiFi
PretextView	0.2	https://github.com/wtsi-hpag/PretextView
purge_dups	1.2.3	https://github.com/dfguan/purge_dups
sanger-tol/genomenote	v1.0	https://github.com/sanger-tol/genomenote
sanger-tol/readmapping	1.1.0	https://github.com/sanger-tol/readmapping/tree/1.1.0
YaHS	1.1a.2	https://github.com/c-zhou/yahs

### Genome annotation

The Ensembl Genebuild annotation system for non-vertebrate species ( Aken *et al.*, 2016) was used to generate annotation for the *Tridacna derasa* assembly (GCA_963210305.1) in Ensembl Rapid Release at the EBI. Annotation was created primarily through alignment of transcriptomic data to the genome, with gap filling via protein-to-genome alignments of a select set of proteins from UniProt ( UniProt Consortium, 2019).

### Wellcome Sanger Institute – Legal and Governance

The materials that have contributed to this genome note have been supplied by a Tree of Life collaborator. The Wellcome Sanger Institute employs a process whereby due diligence is carried out proportionate to the nature of the materials themselves, and the circumstances under which they have been/are to be collected and provided for use. The purpose of this is to address and mitigate any potential legal and/or ethical implications of receipt and use of the materials as part of the research project, and to ensure that in doing so we align with best practice wherever possible. The overarching areas of consideration are:

•   Ethical review of provenance and sourcing of the material

•   Legality of collection, transfer and use (national and international)

Each transfer of samples is undertaken according to a Research Collaboration Agreement or Material Transfer Agreement entered into by the Tree of Life collaborator, Genome Research Limited (operating as the Wellcome Sanger Institute) and in some circumstances other Tree of Life collaborators.

## Data Availability

European Nucleotide Archive: *Tridacna derasa* (giant clam). Accession number PRJEB62732; https://identifiers.org/ena.embl/PRJEB62732 ( Wellcome Sanger Institute, 2023). The genome sequence is released openly for reuse. The *Tridacna derasa* genome sequencing initiative is part of the Aquatics Symbiosis Genomics (ASG) project. All raw sequence data and the assembly have been deposited in INSDC databases. Raw data and assembly accession identifiers are reported in Table 1.

## Author information

Members of the Wellcome Sanger Institute Tree of Life Management, Samples and Laboratory Team are listed here: https://doi.org/10.5281/zenodo.10066175.

Members of the Wellcome Sanger Institute Scientific Operations: Sequencing Operations are listed here: https://doi.org/10.5281/zenodo.10043364.

Members of the Wellcome Sanger Institute Tree of Life Core Informatics team are listed here: https://doi.org/10.5281/zenodo.10066637.

Members of the European Bioinformatics Institute ASG Data Portal team are listed here: https://doi.org//10.5281/zenodo.10076466.

Members of the Wellcome Sanger Institute/Aquatic Symbiosis Genomics Project Leadership are listed here: https://doi.org/10.5281/zenodo.10184833.
