# Supplementary material for: The genome sequence of the smooth giant clam, Tridacna derasa Röding, 1798
Source: Wellcome Open Res. 2024 Jul 16;9:375. [Version 1] doi: 10.12688/wellcomeopenres.22618.1 (PMC11490831; doi:10.12688/wellcomeopenres.22618.1)
Supplement: Peer review history

# Reviewer report: Reviewer response for version 1

**DOI:** 10.21956/wellcomeopenres.24918.r102719

**Published:** 2024-11-18

**Authors:** Ziniu Yu, Yang Zhang

**Affiliations:** 1 South China Sea Institute of Oceanology, Chinese Academy of Science, Guangzhou, China

This paper focus on genome sequence of the smooth giant clam (Tridacna derasa). The author successfully assembled the genome sequence of T. derasa by using the cutting-edge sequencing technology such as HIFI and Hi-C, and a proven assembly and annotation pipeline. The genome has a total length of 1.06 Gb in 21 sequence scaffolds with a scaffold N50 of 63.7 Mb, most of the assembly sequence (99.83%) was assigned to 18 chromosomal-level scaffolds, proving the integrity and accuracy of the genome sequence, which is a good work in the shellfish genome assembly.

 This study not only provides a basis for the study of the population genetics of T. derasa, but also reflects valuable insights into the study of genome evolution of giant clams.

 However, I would suggest that add “of” after the “individual” in the first sentence of the abstract. And I believe that the result for evaluating the completeness of the assembly by BUSCO v5.3.2 should add up to 100%, please check it. In addition, what accounts for the slightly higher percentage of missing BUSCOs (17.8%) in the evaluation of genome assembly quality? A little bit explanation is needed here.

Are sufficient details of methods and materials provided to allow replication by others?

Yes

Is the rationale for creating the dataset(s) clearly described?

Yes

Are the datasets clearly presented in a useable and accessible format?

Yes

Are the protocols appropriate and is the work technically sound?

Yes

Reviewer Expertise:

Immunology, genomics

We confirm that we have read this submission and believe that we have an appropriate level of expertise to confirm that it is of an acceptable scientific standard, however we have significant reservations, as outlined above.

# Reviewer report: Reviewer response for version 1

**DOI:** 10.21956/wellcomeopenres.24918.r102127

**Published:** 2024-10-19

**Authors:** Qinggang Xue

**Affiliations:** 1 Zhejiang Wanli University, Ningbo, Zhejiang, China

The article presents the sequencing and assemblage of the genome of the giant clam, Tridacna dears. The work is well justified with its potential in further investigation of the evolution and environmental adaptation of related species. Overall, the presented results indicated a high quality genome assembly. I just have one questions:

 How were the DNAs of the symbiotic Symbiodiniaceae dinoflagellates removed when preparing the clam DNAs for sequencing?

Are sufficient details of methods and materials provided to allow replication by others?

Partly

Is the rationale for creating the dataset(s) clearly described?

Yes

Are the datasets clearly presented in a useable and accessible format?

Yes

Are the protocols appropriate and is the work technically sound?

Yes

Reviewer Expertise:

Bivalve functional genomics

I confirm that I have read this submission and believe that I have an appropriate level of expertise to confirm that it is of an acceptable scientific standard.

# Reviewer report: Reviewer response for version 1

**DOI:** 10.21956/wellcomeopenres.24918.r95045

**Published:** 2024-09-03

**Authors:** Jian-Wen Qiu

**Affiliations:** 1 Department of Biology, Hong Kong Baptist University, Hong Kong, Hong Kong, China

The authors reported the genome sequence of the smooth giant clam Tridacna derasa. The data will help evolutionary studies of giant clams, and contribute to the population genetic studies of T. derasa.

 The sequencing was conducted with state-of-the-art techniques, and the assembly and annotation procedures used had been applied to similar clam genomes. The assembly is of high-quality, as demonstrated by the large percentage of scaffolds anchored to the 18 pseudochromosomes, the low contamination, and the high QV value, although the number of missing BUSCO genes appears high (17.8%).

 Overall, the genome sequence will be useful for studies of molluscan genome evolution and population genetics of this giant clam. The paper is also well-written.

 I found only one minor error in the Abstract - there should have been an "of" between "individual" and "Tridacna".

Are sufficient details of methods and materials provided to allow replication by others?

Yes

Is the rationale for creating the dataset(s) clearly described?

Yes

Are the datasets clearly presented in a useable and accessible format?

Yes

Are the protocols appropriate and is the work technically sound?

Yes

Reviewer Expertise:

Phylogenetics and genomics of marine invertebrates.

I confirm that I have read this submission and believe that I have an appropriate level of expertise to confirm that it is of an acceptable scientific standard.
